# Parametric computation predicts a multiplicative interaction between synaptic strength parameters that control gamma oscillations

**DOI:** 10.3389/fncom.2012.00053

**Published:** 2012-07-24

**Authors:** Jordan D. Chambers, Blair Bethwaite, Neil T. Diamond, Tom Peachey, David Abramson, Steve Petrou, Evan A. Thomas

**Affiliations:** ^1^Florey Neuroscience Institutes, ParkvilleVIC, Australia; ^2^Department of Physiology, The University of Melbourne, ParkvilleVIC, Australia; ^3^Faculty of Information Technology, Monash University, ClaytonVIC, Australia; ^4^Department of Econometrics and Business Statistics, Monash University, ClaytonVIC, Australia; ^5^Centre for Neural Engineering, The University of Melbourne, ParkvilleVIC, Australia; ^6^Department of Anatomy and Neuroscience, The University of Melbourne, ParkvilleVIC, Australia

**Keywords:** parametric computation, cortical network, traub model, persistent gamma, fractional factorial design, microcircuits, chattering neurons

## Abstract

Gamma oscillations are thought to be critical for a number of behavioral functions, they occur in many regions of the brain and through a variety of mechanisms. Fast repetitive bursting (FRB) neurons in layer 2 of the cortex are able to drive gamma oscillations over long periods of time. Even though the oscillation is driven by FRB neurons, strong feedback within the rest of the cortex must modulate properties of the oscillation such as frequency and power. We used a highly detailed model of the cortex to determine how a cohort of 33 parameters controlling synaptic drive might modulate gamma oscillation properties. We were interested in determining not just the effects of parameters individually, but we also wanted to reveal interactions between parameters beyond additive effects. To prevent a combinatorial explosion in parameter combinations that might need to be simulated, we used a fractional factorial design (FFD) that estimated the effects of individual parameters and two parameter interactions. This experiment required only 4096 model runs. We found that the largest effects on both gamma power and frequency came from a complex interaction between efficacy of synaptic connections from layer 2 inhibitory neurons to layer 2 excitatory neurons and the parameter for the reciprocal connection. As well as the effect of the individual parameters determining synaptic efficacy, there was an interaction between these parameters beyond the additive effects of the parameters alone. The magnitude of this effect was similar to that of the individual parameters, predicting that it is physiologically important in setting gamma oscillation properties.

## Introduction

Neuronal activity throughout the hippocampus and cortex is characterized by power in the gamma band (30–80 Hz). Gamma oscillations occur during sleep and wakefulness and have been hypothesized to be involved in attentive sensory processing, working memory and binding of sensory features (Gray, 1994; Singer and Gray, 1995). A recent report has suggested that gamma oscillations may enhance information transmission (Sohal et al., 2009). These oscillations are found in the olfactory bulb (Adrian, 1942), hippocampus (Bragin et al., 1995) and in most regions of the cortex (Bouyer et al., 1981; Eckhorn et al., 1988). They also occur in a number of *in vitro* preparations (Fisahn et al., 1998; Cunningham et al., 2003). The ubiquity and robustness of gamma oscillations suggest that there may be several mechanisms that generate them (Wang, 2010; Whittington et al., 2011). In one mechanism, persistent gamma, oscillations are driven by fast repetitive bursting (FRB) neurons, also known as chattering cells. These oscillations are observed in slices perfused with carbachol or kainate (Buhl et al., 1998; Cunningham et al., 2004). FRB cells are pyramidal neurons which respond to current injections with bursts of high frequency action potentials but with a burst frequency in the gamma range. They are mainly located in superficial layers in the somatosensory cortex (Gray and McCormick, 1996) but may be located in all layers in the visual cortex at least in cat (Cardin et al., 2005). Although FRB neurons play a key role in driving persistent gamma, many neurons in the cortex are recruited into the oscillation. Furthermore, there is likely to be a complex interaction between single FRB neuron properties, the microcircuit of coupled FRB neurons and the larger network.

Exploring mathematical models is an essential part of understanding complex neural systems. These models vary enormously in the amount of detail represented in them. Lumped models, for example, represent activity in a population of neurons with a small number of state variables (Wilson and Cowan, 1972; Lytton, 2008) and the differential equations for the state variables have a correspondingly small number of parameters. The goal of these models is to develop a global view of system behavior over the entire parameter space and this, in turn, provides a complete qualitative understanding of the system. On the other hand, highly detailed models are able to link subtle biophysical changes to system behaviors. Genetic epilepsy is an example of where this is useful (Thomas and Petrou, 2008). Single mutation epilepsies may shift a biophysical parameter, for example voltage dependence of channel activation, by a few millivolts which in turn may have functional consequences (Thomas et al., 2007, 2009). Computer simulation can link these changes to larger network behaviors but the models must incorporate fine grain biophysical detail.

Highly detailed models are parametrically complex and are difficult to analyse for a number of reasons. Firstly, many parameters are uncertain because they are not measured for the particular species, age, brain region, or neuron type being described. Secondly, there are also important differences in preparations or behavioral state of the animal. The resting membrane potential, for example, is different *in vivo* during normal behavior compared to measurements *in vitro* such as in brain slices (Destexhe et al., 2001). If the behavior of interest is thought to be sensitive to an uncertain parameter then the modeler needs to vary the parameter in order to determine whether it is relevant or not. For large models there will be many uncertain parameters and the combinatorial explosion in the number of parameter combinations prevents an exhaustive examination of the entire parameter space. Typically this is dealt with in an *ad hoc* manner with only a small number of parameters varied. This not only misses potentially important parameters but may also miss unexpected interaction between parameters. Another problem with *ad hoc* methods is that parameter exploration is done around a base or standard configuration of the model, usually chosen because it is either stationary or reproduces a behavior of interest. Thus, *ad hoc* exploration provides only a limited view of the parameter space in the local region around the base parameter set.

It will never be possible to fully analyse a parametrically complex, non-linear model. However, with reasonable assumptions and by reacting to model output it is possible to generate a global view of large parameter spaces. The first assumption is that a realistic model is ‘well behaved’ in that response values are smooth between physiological parameter values if there is no bifurcation. If a bifurcation is present parameter values can be narrowed to a region with smooth behavior. This same assumption is made in experimental studies, for example when two drug concentrations or two values of a stimulus are applied to a biological preparation. The second assumption is that interactions between large numbers of parameters are small. Specifically, this means responses beyond the cumulative effect of individual parameter changes are small for large numbers of parameters. Again, this is also an assumption of biological experiments were only a small number of parameters can be varied. With these two assumptions it is possible to develop formal methods for handling large numbers of parameters.

In this paper, we apply a method known as fractional factorial design (FFD) (Box et al., 2005) to a detailed model of the thalamocortical network. The method generates a subset of a full factorial design (i.e., all parameter combinations), usually a significantly smaller subset. The outputs of the model are then fitted to a response surface which estimates the effects of parameters and parameter combinations. In the designs used here, the primary effects of parameters are determined and Two-Way parameter interactions, beyond the additive effects of individual parameters. The model that we used is a previously published highly detailed model of the thalamocortical network (Traub et al., 2005b), one of the most detailed neural network models published (Kopell, 2005). This network displays a variety of behaviors including gamma oscillations. In this study, we concentrate on gamma-like activity and examine the role of 33 parameters determining synaptic strength. The number of runs required to examine two parameter values using a full factorial design is 2^33^ or approximately 8.6 × 10^9^ which is not tractable. Using the FFD software in the Nimrod grid toolkit (Peachey et al., 2008a) we generated a design consisting of 4096 runs which is manageable on a high performance computing facility.

## Materials and methods

### Parametric computation

Experimental design has been a formal science for nearly a 100 years (Fisher, 1926). The techniques have gradually spread from agricultural science to other sciences and to industrial process control, but until recently, little use has been made in experiments involving computer models. Our work uses one of the fundamental design methods, FFD. Since this seems to be the first application of FFD in computational neuroscience, we offer an introduction to the area. A full description may be found in Box et al. (2005); applications to computer modeling are described in Peachey et al. (2008a) and Sher et al. (2010).

We consider a model with *n* parameters *a, b, c, etc*. and response, ϕ. Any such integrable function may be expanded as

(1) $$\phi =k+\{\psi_{1}(a)+\psi_{2}(b)+\ldots \}+\{\psi_{12}(a,b)+\psi_{13}(a,c)+\psi_{23}(b,c)+\ldots \}+\{\psi_{123}(a,b,c)+\ldots \}+\ldots$$

Where *a, b, c, etc*., are parameters, *k* is the mean response averaged over all parameter inputs, ψ_1_(*a*) is the deviation from *k* due to *a*, averaged over all values of the other parameters, ψ_12_(*a, b*) is the deviation from *k* due to interaction between *a* and *b*, averaged over *c, d*,…, and so on. The functions ψ_*i*_ are known as “main effects”, ψ_*ij*_ are “Two-Way interaction effects”, ψ_*ijk*_ are “three-way interactions” and so on. Although Equation (1) is not a Taylor expansion (each ψ may be a complicated, even discontinuous, function) it shares the property that, for all practical examples, the high order interactions are relatively small and may be ignored.

Numerical explorations of the model must, of course, be limited to discrete parameter sets. The simplest useful such search uses just two values for each input parameter, a low and a high value. We assume that these parameters are translated and scaled so that the high values all equal 1 and the low values –1, thus giving every parameter an equal weight in what follows. Now the effect ψ_1_(*a*), for example, can take only two values. These must be of the form −*k*_*a*_, +*k*_*a*_ since their mean is zero, so ψ_1_(*a*) may be written as *k*_*a*_ *a*. Similar considerations apply to all effects, and equation (1) reduces to

(2) $$\phi =k+\{k_{a}a+k_{b}b+k_{c}c+\ldots \}+\{k_{ab}ab+k_{ac}ac+k_{bc}bc+\ldots \}+\{k_{abc}abc+\ldots \}+\ldots$$

With a slight abuse of terminology, these various *k* are also called effects. There are 2^*n*^ of them, so evaluation of ϕ for all combinations of the parameter values, a “parameter sweep” or a “full factorial experiment,” will suffice to evaluate them.

If the actual response function is discontinuous, perhaps due to bifurcations in the underlying phenomena, then (2) cannot give an accurate approximation over the whole space. But even in such difficult cases, practice shows that the absolute values of the effects can still indicate the relative importance of the various parameters and interactions in producing the final response. The sign of each effect gives the direction of the contribution.

Results will not be definitive with such a coarse grained sampling. For example if ψ_1_(*a*) is actually *a*^2^ then evaluation at *a* = ± 1 will show *k*_*a*_ = 0, missing a possibly important effect. Errors such as these are errors of omission rather than false positives. Further work with different values for the parameters or with a finer grained model may reveal the missing effect. As in all scientific investigations, results are indicative rather than definitive.

On the subject of finer grained models, the next most common model would use four values for each parameter. It requires two subsidiary variables of ±1 for each parameter, effectively representing the two bits in the specification. This would then reveal quadratic and cubic terms in the model but vastly increases the computational load and makes interpretation much harder.

The determination of all the effects via a parameter sweep is not practical when *n* is large; our case of 33 parameters would require 2^33^, over eight billion, runs of the model. However, even when a large number of parameters are involved, typically just a handful of the low order effects are significant and suffice to produce an accurate approximation to ϕ. Because of this, it is possible to greatly reduce the required number of runs using FFDs where a carefully chosen subset of the full factorial design will suffice to determine the lower order effects. The crux of the design is to find a “defining contrast,” where products of the parameters are constrained to the value 1. Consider the constraint *acde* = 1. Given that each parameter may only take values ±1, once values for *a*, *c* and *d* are chosen, the value for *e* is predetermined, so the total number of runs possible is halved. There is a price to pay, however; now *ac* will always take the same value as *de* so it becomes impossible to distinguish the two effects, an estimate is only possible for the combination *k*_*ac*_ + *k*_*de*_. We say that *k*_*ac*_ is “biased” by *k*_*de*_, and *vice versa*. Similarly, *k*_*ad*_ is biased by *k*_*ce*_, *k*_*a*_ is biased by *k*_*cde*_, *k* by *k*_*acde*_, and so on. Clearly this is an unacceptable constraint to use. A longer string in the constraint would bias low order effects only against negligible ones. However, when several constraints are used, they may combine to give undesirable biases. We require a “resolution V” design, that is, one that biases second order effects against third order ones at the worst, and first order effects against fourth order. Note that once a suitable design has been found it may be used for all the outputs of interest from the model; there is no need to recompute the parameter set for each output.

The design of FFDs is a difficult task. Usually, experimenters consult literature or a web site for a suitable design. Such resources do not supply the very large designs needed for some computational experiments. Recently, (Peachey et al., 2012), building on work by Liao and Iyer (1999), have developed an algorithm that for the first time can produce designs of resolution V with up to 130 parameters. The algorithm has been incorporated in the Nimrod/E software (Peachey et al., 2008a) and is freely available for download and use (MessageLab http://www.messagelab.monash.edu.au/). Different defining contrasts, for the same design requirement, will change the precise combination of higher order effects biasing lower order effects but not the order of the biases. Under the assumption that higher order effects are insignificant different designs will not be significantly different from each other. Our software created 21 constraints for a 33 parameter experiment and produced 2^33−21^ = 4096 runs.

The fractional experimental design including the defining contrast and other source files required to reproduce our experiments are available at ModelDB or by contacting the authors.

Only a handful of effects typically have a statistically significant influence on model response and most effects are the result of cumulative, small influences on the output and thus normally distributed. To visualize significant effects, effects are plotted against their quantiles together with a line indicating an ideal normal distribution. Effects for normal plots in Figures 5A and 6A and significance values in Figures 5B and 6B were estimated using the Yates algorithm (Box et al., 2005). Effects for Figures 5B and 6B were calculated by fitting Equation (2), truncated after second order effects, to the output. This is a linear model in the effects and fitting was done by standard linear regression. The contribution to the variance in Figures 5C and 6C was calculated by summing the square of the effects, where effects are twice the size of the regression coefficients.

We also estimated the quadratic effects for the three most influential parameters. As discussed later, we had also run a full factorial experiment in which the three most influential parameters were varied over four values each, ranging from half the midpoint value to twice the midpoint value, with the other parameters being set to midpoint values. This design allows estimation of the model

(3) $$\phi =k+\{k_{a}a+k_{b}b+k_{c}c\}+\{k_{ab}ab+k_{ac}ac+k_{bc}bc\}+\{k_{aa}a^{2}+k_{bb}b^{2}+k_{cc}c^{2}\}$$

where the three most influential parameters have been labeled *a, b*, and *c*.

### The cortical model

**Figure 1:**
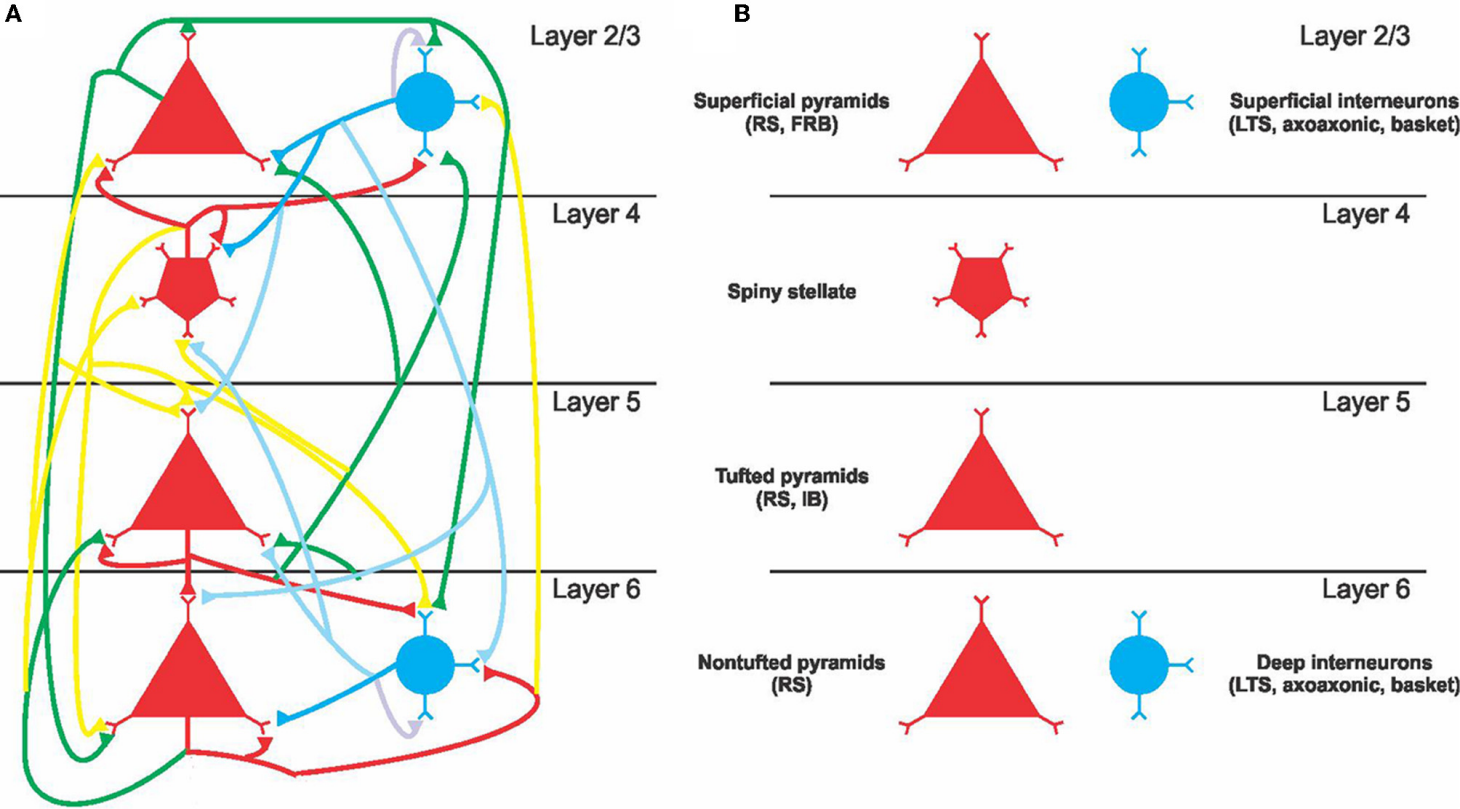
**Simplified diagram of the cortical network. (A)** Layer structure and connectivity between excitatory and inhibitory neurons. The color of the lines indicates the strength of synaptic connection: red for strong excitatory, green for intermediate excitatory, yellow for weak excitatory, dark blue for strong inhibitory, and light blue for weak inhibitory. **(B)** Neuron classes represented in the model.

**Figure 2:**
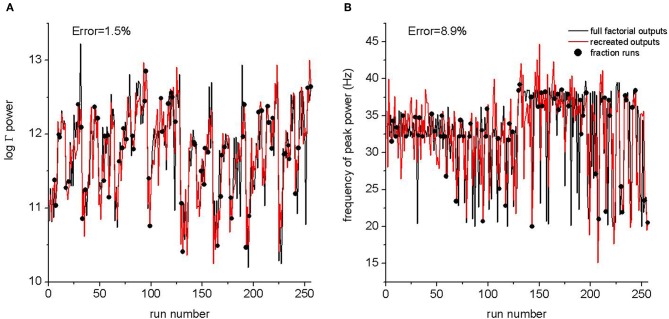
**A comparison between the full factorial design of 256 runs and the response predicted from a fractional design of 64 runs.** The run number is an arbitrary assignment to runs from the full factorial experiments. Outputs from the fractional experiment are plotted as black circles with the run number corresponding to the parameter values from the full experiment. The black line is the output from the full experiment. The red line is the output of Equation 2 after effects were estimated from the fractional experiment. The error is the normalized mean of the difference between the recreated output and the output from the full experiment. **(A)** Log of the mean power in the 20–80 Hz power band. **(B)** Frequency of peak power. The relative error between the full sweep and the fractional sweep are indicated.

A full description of the model is provided in Traub et al. (2005b). Method validation (Figure 2) was performed with the full model and the large parameter space experiment was performed without thalamic neurons. The model contains the following neuron types: layer 2/3 rhythmic spiking pyramidal cell (*suppyrRS*); layer 2/3 fast rhythmic bursting (FRB) pyramidal cell (*suppyrFRB*); superficial basket cell (*supbask*); superficial axoaxonic cell (*supaxax*); layer 2/3 low threshold spiking interneuron (*supLTS*); layer 4 spiny stellate cell (*spinstell*); layer 5 tufted intermediate bursting pyramidal cell (*tuftIB*); layer 5 tufted rhythmic spiking pyramidal cell (*tuftRS*); layer 6 non-tufted rhythmic spiking pyramidal cell (*nontuftRS*); deep basket cell (*deepbask*); deep axoaxonic cell (*deepaxax*); deep low threshold spiking interneuron (*deepLTS*); and the following synapses: AMPA, NMDA, and GABA_A_; Conductances: (1) A fast sodium conductance for action potential generation, concentrated in the soma with dendritic density decreasing distally, (2) a fast delayed rectifier potassium conductance, responsible for action potential repolarization and following the sodium channel density distribution (3) a persistent Na conductance on the soma and less densely on the dendrites, (4) a high voltage activated calcium conductance (L type) uniformly distributed, (5) a low voltage activated calcium conductance (T type) similarly distributed, (6) a calcium and voltage activated potassium current (BK channels) with a density following the calcium conductance, (7) a calcium, but not voltage, activated potassium conductance (AHP) also following the calcium conductance distribution, (8) a non-inactivating potassium current (M current) uniformly distributed, (9) a rapidly inactivating potassium current (A current) present on the soma and proximal apical dendrite and to a lesser extent on other dendrites, (10) a slowly inactivating potassium current (K2 current), uniformly distributed on the membrane and (11) a hyperpolarization activated inward current (h current) with density increasing distally. Conductance densities were the same as Traub et al. (2005b) (note this differs from the codes available in ModelDB). A diagram of the neuron and synapse types is presented in Figure 1.

FRB neurons received constant random current injections to mimic the effect modulators that drive activity in these neurons. These neurons fired bursts of action potentials with a burst frequency in the gamma range (Cunningham et al., 2004; Traub et al., 2005a).

The specific parameters that were varied as part of this study were post synaptic conductance densities (Table 1). Inhibitory synapses are controlled by the density of GABA_A_ receptors and excitatory synapses are controlled by both the density of AMPA and NMDA receptors which were scaled together. In this study, we varied synaptic efficacy between populations of neurons defined as excitatory or inhibitory populations in layers 2/3, 4, 5, and 6. Parameter names reflect these populations of synapses, for example *e5i2* represents the group of synapses from all excitatory layer 5 neurons to all inhibitory layer 2 neurons. Thus, the parameter values analysed here map to several conductance values in the model. Low values of each parameter were half that in the original paper and high values were twice the original value. Parameters in terms of individual synapses are provided in our version of the model available in ModelDB.

**Table 1:** **Model parameters varied in this study**.

**Parameter**	**Source**	**Target**	**Parameter**	**Source**	**Target**
e2e2	suppyrFRB	suppyrFRB	e2e5	suppyrFRB	tuftIB
	suppyrFRB	suppyrRS		suppyrFRB	tuftRS
	suppyrRS	suppyrFRB		suppyrRS	tuftIB
	suppyrRS	suppyrRS		suppyrRS	tuftRS
e2e6	suppyrFRB	nontuftRS	e2i2	suppyrFRB	supLTS
	suppyrRS	nontuftRS		suppyrFRB	supaxax
e2i6	suppyrFRB	deepLTS		suppyrFRB	supbask
	suppyrFRB	deepaxax		suppyrRS	supLTS
	suppyrFRB	deepbask		suppyrRS	supaxax
	suppyrRS	deepLTS		suppyrRS	supbask
	suppyrRS	deepaxax	e4e2	spinstell	suppyrFRB
	suppyrRS	deepbask		spinstell	suppyrRS
e4e4	spinstell	spinstell	e4e5	spinstell	tuftIB
e4e6	spinstell	nontuftRS		spinstell	tuftRS
e4i6	spinstell	deepLTS	e4i2	spinstell	supLTS
	spinstell	deepaxax		spinstell	supaxax
	spinstell	deepbask		spinstell	supbask
e5e2	tuftIB	suppyrFRB	e5e4	tuftIB	spinstell
	tuftIB	suppyrRS		tuftRS	spinstell
	tuftRS	suppyrFRB	e5e5	tuftIB	tuftIB
	tuftRS	suppyrRS		tuftIB	tuftRS
e5e6	tuftIB	nontuftRS		tuftRS	tuftIB
	tuftRS	nontuftRS		tuftRS	tuftRS
e5i6	tuftIB	supLTS	e6e2	nontuftRS	suppyrFRB
	tuftIB	supaxax		nontuftRS	suppyrRS
	tuftIB	supbask	e6e4	nontuftRS	spinstell
	tuftRS	supLTS	e6e6	nontuftRS	nontuftRS
	tuftRS	supaxax	e6i2	nontuftRS	supLTS
	tuftRS	supbask		nontuftRS	supaxax
e6i6	nontuftRS	deepLTS		nontuftRS	supbask
	nontuftRS	deepaxax	i2e2	supLTS	suppyrFRB
	nontuftRS	deepbask		supLTS	suppyrRS
i2e4	supLTS	spinstell		supaxax	suppyrFRB
	supaxax	spinstell		supaxax	suppyrRS
	supbask	spinstell		supbask	suppyrFRB
i2e5	supLTS	tuftIB		supbask	suppyrRS
	supLTS	tuftRS	i2i6	supLTS	deepLTS
	supaxax	tuftIB		supLTS	deepaxax
	supaxax	tuftRS		supLTS	deepbask
i2e6	supLTS	nontuftRS	i6e4	deepLTS	spinstell
	supaxax	nontuftRS		deepbask	spinstell
i2i2	supLTS	supLTS	i6e5	deepLTS	tuftIB
	supLTS	supaxax		deepLTS	tuftRS
	supLTS	supbask		deepaxax	tuftIB
	supbask	supLTS		deepaxax	tuftRS
	supbask	supaxax		deepbask	tuftIB
	supbask	supbask		deepbask	tuftRS
i6i6	deepLTS	deepLTS	i6e6	deepLTS	nontuftRS
	deepLTS	deepaxax		deepaxax	nontuftRS
	deepLTS	deepbask		deepbask	nontuftRS
	deepbask	deepLTS		
	deepbask	deepaxax			
	deepbask	deepbask			

Each parameter determines conductance density at the corresponding synapses. A parameter value of −1 corresponds to half the value in the original model and a value of +1 corresponds to twice the original value.

An approximation to the extracellular field potentials (EFPs) was calculated as follows (Traub et al., 2005b); each neuron type has an assigned depth based on anatomical data for rat auditory cortex. EFP at the recording site is proportional to the sum of currents generated by the soma and basal and apical dendrites of pyramidal neurons. Assuming constant extracellular resistivity this will be proportional to the product of the transmembrane voltage and the compartment surface area and inversely proportional to the distance to the recording electrode. Because no specific values of the extracellular resistivity or membrane conductance were used, EFP units are arbitrary. We calculated the EFP for an electrode located on the surface of the cortex representing an electrocorticogram.

Power spectra were estimated from the last 1000 ms of the run, to avoid influence from the initial conditions, using a periodogram and band passed as indicated in the text.

### Computational details

Simulations were performed with the NEURON simulation software (Carnevale and Hines, 2006) using a port of the original FORTRAN code (available from ModelDB). A parallel version of the code was generously provided by Michael Hines. This was parametrically identical to the serial version and our testing confirmed that that the parallel and serial version produced the same results. The parallel version of the code performed satisfactorily up to 40 processors, the maximum number we tested. Runs were 2000–3000 ms and each took 20–30 CPU h on an Intel 2.7 GHz Xeon processor. Experimental design and run management was performed by the Nimrod tool chain (messagelab.monash.edu.au/Nimrod). Effect estimation and statistical analysis was performed by custom Matlab (Mathworks, USA) and R scripts (www.r-project.org).

## Results

### Method validation

We tested the ability of a FFD to estimate the response of a full factorial design. We studied eight parameters that scaled all synaptic output from inhibitory or excitatory neurons for each of layers 6, 5, 4, and 2/3 independently of target population. A full experiment requires the model to be run for each parameter combination for each of two values per parameter, that is 2^8^ = 256 runs. We also designed a 2^8−2^ = 64 fractional factorial run that estimated first and second order effects possibly biased by third or higher order effects. This experiment was designed using Nimrod/E software (Peachey et al., 2008a) which produced the defining contrast *bcdgh* and *acdef*. The complete design of the experiment is available as part of the ModelDB submission. Figure 2 compares output from the full factorial design to output recreated after estimating effects from the FFD. The outputs that we tested were total power and frequency of peak power in the 20–80 Hz band. The fractional experiment was able to reproduce the peak frequency within 9%, and the log total power to within 2%. This is more than adequate given other uncertainties in biological models.

### Model output

**Figure 3:**
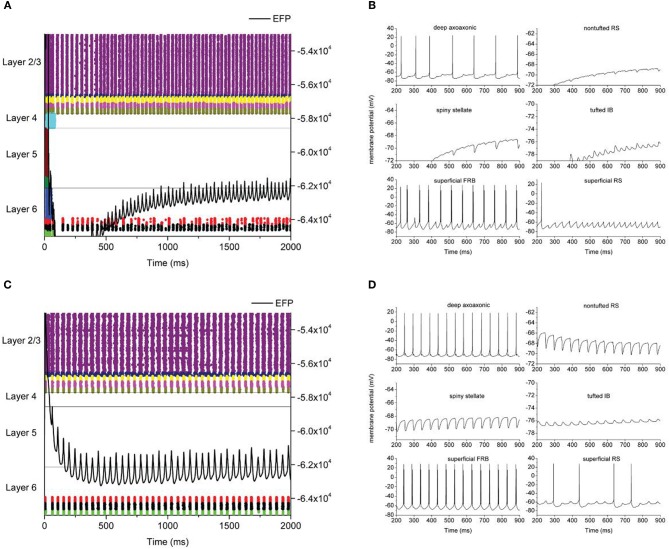
**Example model output.** Left hand panels **(A,C)** are raster plots where each dot represents an action potential with neuron types color coded and cortical layers as indicated. Neuron types are, from top to bottom, Layer 2/3: superficial RS, superficial FRB, superficial basket, superficial axoaxonic, superficial LTS. Layer 4: spiny stellate. Layer 5: tufted IB, tufted RS. Layer 6: nontufted RS, deep basket, deep axoaxonic, deep LTS. The solid line is local field potential with magnitude indicated on the right hand axis. Right hand **(B,D)** panels are membrane potential traces from individual neurons as indicated.

**Figure 4:**
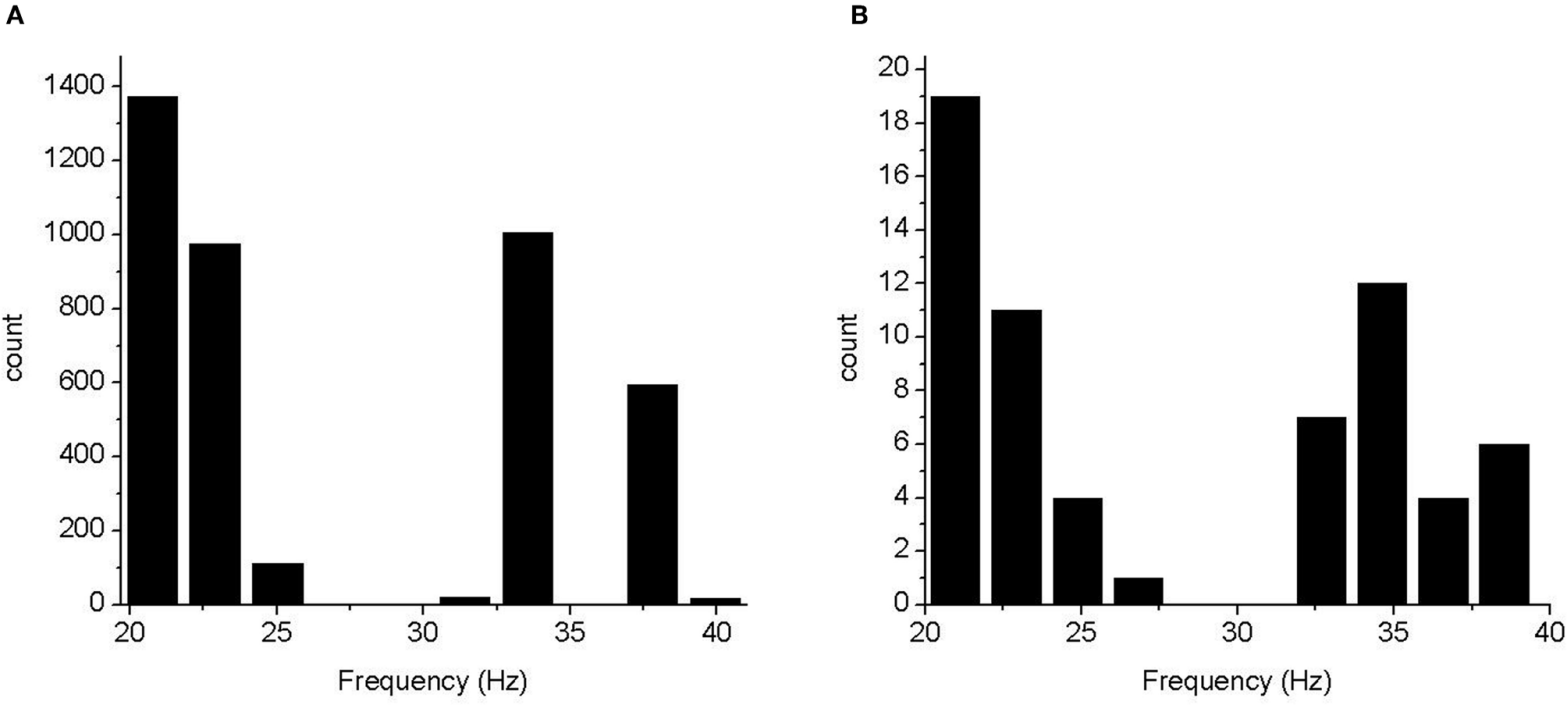
**Histograms of frequency of peak power. (A)** Frequencies from the 4096 run experiment. **(B)** Frequencies from an experiment in which the three most influential parameters were varied over four levels each.

We applied the FFD to investigate how the synaptic connections between the different layers of a cortical column influence gamma oscillations. All 4096 runs for the model displayed robust oscillations in the 15–80 Hz range. Example raster plots and single neuron membrane potential traces are shown in Figure 3. The oscillation frequency was defined as the frequency of peak power in 15–80 Hz band. A histogram of the frequency distribution is shown in Figure 4. As a control, to demonstrate that FRB neurons are driving the oscillation, we were able to quench the oscillation when negative current was injected into FRB neurons.

All neuron types in layer 2/3 were consistently recruited into the oscillation. Layer 4 was consistently silent, neurons failed to fire action potentials and were subject to heavy inhibitory synaptic bombardment (Figure 3). Layer 5 neurons were also silent but did receive excitatory synaptic bombardment from superficial layers. Layer 6 inhibitory neurons were recruited into the oscillation, however, the degree of recruitment varied between runs (Figures 3A vs. 3C). These neurons fired action potentials slightly behind superficial cortical activity, but otherwise synchronized with the oscillation. Layer 6 pyramidal neurons were subject to inhibitory synaptic bombardment from layer 6 inhibitory neurons.

### Analysis of run output

We analysed the peak frequency and power at peak frequency from power spectra derived from the local field potential after the model reached a stable oscillation.

#### Frequency of peak power

The frequency distribution (Figure 4) fell into several distinct bands, some of which were below the definition of gamma frequency. However, in all cases there was power in the gamma band. To determine whether the apparent discretisation of the peak frequency was due to the small number of parameter levels used we performed a full factorial, 64 run, experiment using 4 values of parameters *e2e2*, *e2i2*, and *i2e2*. In this case, the frequency histogram was largely continuous in the 20–40 Hz band (for 15–80 Hz band passed spectra). This indicates that there was no bifurcation in the model over the parameter range tested and the assumption that model varies monotonically over this range is reasonable.

**Figure 5:**
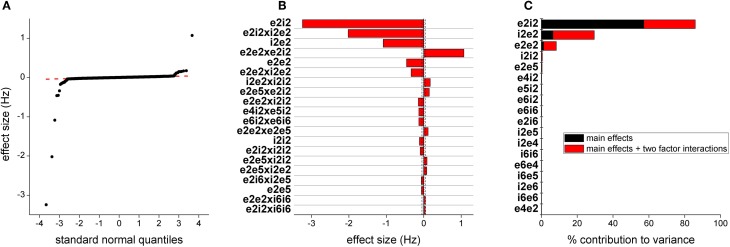
**Parameters determining gamma frequency. (A)** Distribution of effect sizes plotted against quantile value for a normal distribution. **(B)** The top 20 effects by magnitude. The first 2 characters of the parameter labels indicate source neuron population, e/I being inhibitory/excitatory and layer and the second two characters indicate target neuron population. The dashed vertical line indicates 95% confidence and the dashed line indicates 97.5% confidence. **(C)** This bar plot show contribution to the variance in model output. Black indicates the contribution of the parameter alone and red indicates contribution of the parameter as part of a higher order interaction.

We estimated the effects for peak power outputs in the 30–80 Hz band from the 4096 run parameter sweep. A quantile plot of the effect sizes is shown in Figure 5A. Most effects are distributed on a normal distribution, indicated by the straight red line, suggesting that cumulatively they only contribute to noise. The top 25 effects are plotted in Figure 5B together with lines indicating 95 and 97.5% confidence levels. The largest effect is the main effect for *e2i2* which scales synaptic efficacy from both populations of layer 2/3 excitatory neurons to all three populations of layer 2/3 inhibitory neurons. The next largest effect is *e2e2* which scales the efficacy of synaptic transmission from layer 2/3 excitatory neurons onto themselves. The fifth largest effect is *i2e2* which scales synaptic efficacy from all layer 2/3 populations of inhibitory neurons to both populations for layer 2/3 excitatory neurons. The 3rd, 4th, and 6th largest effects are each of the Two-Way interactions, beyond additive, of the three parameters.

As another measure of the influence of parameters on model output, we plotted the contribution to the variance of parameters, either as main effects or in higher order interactions (Figure 5C). The top three effects were the same as those identified through direct estimation of the effects. Synaptic drive in deep layers also contributed to the variance but at much lower levels.

### Power in the gamma band

**Figure 6:**
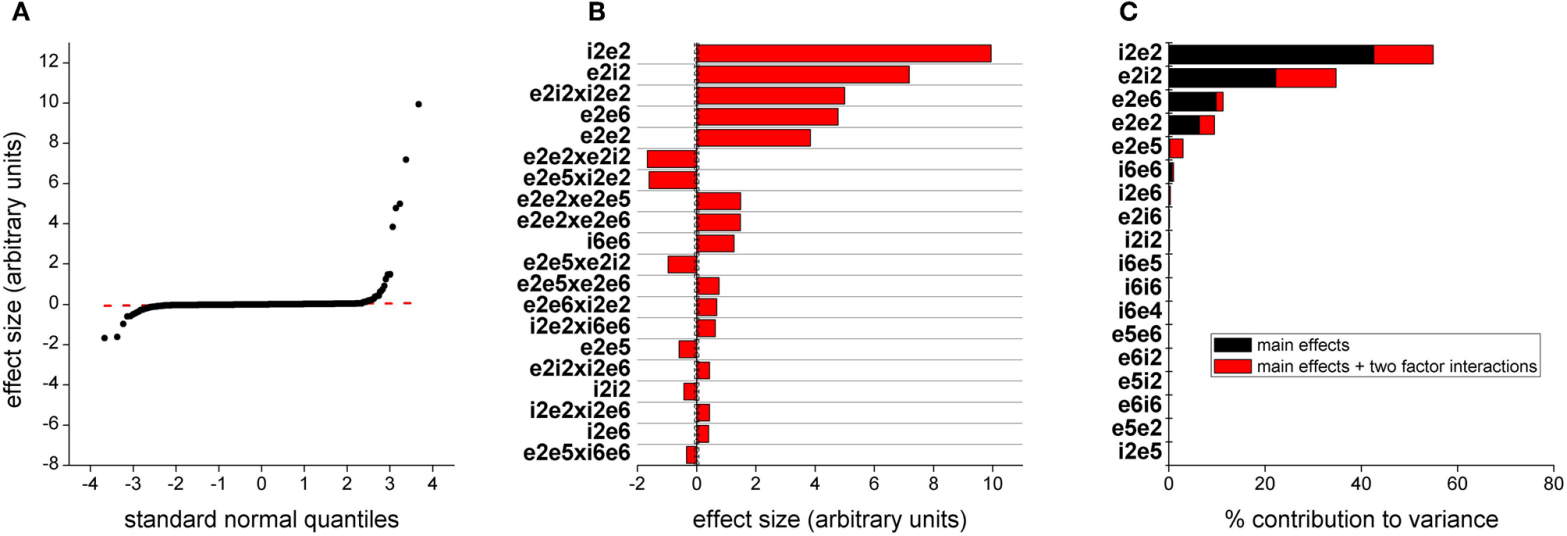
**Parameters determining gamma power. (A)** Distribution of effect sizes plotted against quantile value for a normal distribution. **(B)** The top 20 effects by magnitude. The first 2 characters of the parameter labels indicate source neuron population, i/e being inhibitory/excitatory followed by layer number and the second two characters indicate type and location of target neuron population. The dashed vertical line indicates a 95% confidence and the dashed line indicates 97.5% confidence. **(C)** This bar plot show contribution to the variance in model output. Black indicates the contribution of the parameter alone and red indicates contribution of the parameter as part of a higher order interaction.

We generated model responses from the total power in the 30–80 Hz bands and performed a similar analysis to that in the previous section. Quantile plots of effects are shown in Figure 6A. Most effects fall on a normal distribution and thus are noise. The 25 effects with the largest magnitudes are plotted in Figure 6B together with 95 and 97.5% confidence lines. For these outputs, the largest effects are synaptic drive into layers 5 and 6. The largest effect is the main effect for parameter *e2e5*, scaling synaptic drive from both populations of layer 2/3 excitatory neurons onto both populations of layer 5 pyramidal neurons (Figure 1). The remaining effects are either primary effects for synaptic efficacy between both excitatory and inhibitory neurons within layers 5 and 6 or interactions between these parameters beyond additive. We also determined the contribution of parameters to the variance, either as main effects or as part of higher order interactions (Figure 6C). This analysis also recapitulates the ranking of parameters derived from the direct estimate of effects.

### Estimating quadratic effects

The three parameters with the most influence on gamma frequency are *e2e2, e2i2*, and *i2e2*. Using the output of the 64 run experiment, where each parameter was varied over four values, we fitted the model described by Equation 3 to these data using least squares (Table 2). The regression coefficients show that there is a marked significant interaction between *e2i2* and *i2e2* for both gamma power and frequency of peak power. We also tested a model of the form

(4) $$\phi =k+\{k_{ab}^{\left(1\right)}(a+b)+k_{bc}^{\left(1\right)}(b+c)+k_{ac}^{\left(1\right)}(a+c)\}+\{k_{ab}^{\left(2\right)}{\left(a+b\right)}^{2}+k_{bc}^{\left(2\right)}{\left(b+c\right)}^{2}+k_{ac}^{\left(2\right)}{\left(a+c\right)}^{2}\}$$

**Table 2:** **Effects for a quadratic model**.

	**Power**	**Peak frequency**
k	101292^***^	38.51^***^
k_a_	22402^***^	1.33^*^
k_b_	56794^***^	−6.03^***^
k_c_	66238^***^	−2.18^**^
k_aa_	20535	−0.37
k_bb_	10320	1.88
k_cc_	−5458	−0.10
k_ab_	−14355	1.57
k_ac_	−19208^*^	−0.20
k_bc_	78082^***^	−6.10^***^

Effects estimated for Equation (3) from a 64 run experiment and significance values for linear, quadratic, and interaction terms estimated from cubic terms. Here a, b, and c refer to e2e2, e2i2, and i2e2, respectively.

* , 0.05;

** , 0.01;

*** , 0.001.

However, this model was significantly worse for both gamma power [*F*_(4, 54)_ = 3.20, *p* < 0.020] and for frequency of peak power [*F*_(4, 54)_ = 6.17, *p* < 0.001].

## Discussion

We have studied gamma oscillations in a detailed model of cortical circuitry. The oscillations we studied are driven by so called chattering neurons, pyramidal neurons that fire short bursts of action potentials at approximately gamma frequency when stimulated with current injections or sensory input (Cunningham et al., 2004; Cardin et al., 2005). In the Traub, model these neurons are in layer 2 of the cortex although they may be found in other cortical layers for some regions or species (Cardin et al., 2005). They are nevertheless embedded in a network with a high level of positive and negative feedback through inhibitory and excitatory synaptic connections. The question addressed in this study, is how does synaptic efficacy in this network affect properties of gamma oscillations. We grouped several synaptic parameters into a single parameter controlling synaptic efficacy from excitatory or inhibitory neurons grouped by cortical layer. This created 33 parameters.

### Methodological issues

It is not possible to perform an exhaustive examination of this parameter space. *Ad hoc* methods, such as varying a single or small number of parameters simultaneously, are unsatisfying because they lack rigor and they provide no quantification of parameter interactions. FFD allows the experimenter to determine which potential parameter interactions are detected and the degree to which they may be biased by other interactions. As part of this process the full response of the model can be estimated (Equation 2) and we used this to test the method on a small parameter space (Figure 2). These are important advantages of FFD and other formal methods over *ad hoc* approaches. We believe adoption of formal methods in computational neuroscience will be critical for the study and interpretation of parametrically complex models. To examine our parameter space, we generated a FFD that estimated unbiased first order effects and second order effects biased by higher order effects. This experiment required 4096 runs.

The model described by Equation (2) is linear in each parameter. This model allowed determination of the most significant parameters but does not exclude the possibility of nonlinear effects. Once important parameters are identified, focused experiments can be designed to seek higher order interactions. Using the model described by Equation (3) we tested for quadratic effects in the three most influential parameters identified from the linear model. We found no significant quadratic effects. Interestingly, the new experiment also identified the interaction between *i2e2* and *e2i2* as being statistically significant. The demonstrates how a large experiment, examining many parameters, can be part of a methodology whereby significant effects can be followed up by more directed and smaller experiments.

### Physiological implications

An initial observation is that excitatory neurons in cortical layers 4, 5, and 6 did not spike during the oscillation although they did receive prominent synaptic input (Figures 3B,D). This is potentially significant as it will determine how these neurons process extrinsic inputs. For example, layer 4 neurons receive sensory input directly from the thalamus (Chmielowska et al., 1989) and the response of these neurons to sensory events is highly labile (Petersen et al., 2001). Therefore, a gamma oscillation that produces changes in membrane potential (but not spikes) in layer 4 could be very important in determining the response of the cortical column to sensory input. Pyramidal neurons in layer 5 also receive direct sensory input (Reyes and Sakmann, 1999) as well input from within the column and extra-columnar inputs (Lubke and Feldmeyer, 2007). Layer 6 pyramidal neurons are only weakly innervated by thalamic afferents but have inputs from layer 6 neurons in other columns (Mercer et al., 2005). Response in these neuron populations will be influenced by the membrane potential oscillations. This, in turn, will determine how these neurons process sensory input arriving from the thalamus and inter-columnar input.

We calculated EFPs in the superficial layers for the cortex mimicking the field potential measured by an electrode placed on the surface of the cortex. From the time domain field recordings, we calculated power spectra to determine total power in the gamma band and frequency of peak power in the gamma band. From these outputs we estimated the effects, parameters in Equation 2. The effects are calculated for rescaled, and hence dimensionless, parameter values. Therefore, the relative magnitude of the effects determines how much that parameter, or parameter interaction, influences the output over the full physiological range of the parameter. It is analogous to a sensitivity measure but applies globally rather than at around a single point. In the case of peak frequency, the six largest effects all control synaptic drive within layer 2. For example, the largest effect is the main effect for synaptic efficacy from excitatory layer 2 pyramidal neurons to inhibitory layer 2 neurons (*e2i2*). In this case, the effect has a negative sign indicating that an increase in the value of this parameter decreases the frequency. The synaptic efficacy of the reciprocal connection, that is inhibitory layer 2 neurons to layer 2 excitatory neurons (*i2e2*) also has a large effect on output frequency, with larger values decreasing the frequency of peak power. Interestingly, these parameters had a strong multiplicative interaction. The second largest effect is from the product of these two parameters, *i2e2* × *e2i2*. For example, individually parameters *e2i2* and *i2e2* had effects of −3 and −2, respectively, but the combination of *e2i2* × *i2e2* had an additional effect of –1.2 beyond the additive interaction alone. It is unlikely that this interaction would have been detected by an informal parameter space search.

There was also a contribution to field potential power from the parameter determining excitatory drive from layer 2 into layer 6 (*e2e6*). The deep layers did not fire action potentials, however, synaptically driven membrane potential changes contribute to EFP and this in turn contributes to gamma power.

## Conclusions

Biological systems are parametrically complex, and it remains an open question as to which components of the system are critical for function and which components are constrained by other factors. For this reason, it is important to work with parametrically complex systems just as it is important to work with more abstract models. The strength of abstract models is that they can provide a global understanding of system behavior while the traditional weakness of parametrically complex models is that they only provide local insight into model behavior near the parameter values tested or at best in a small subspace of the entire parameter space. We have used FFD to explore a 33 dimension parameter subspace. Under reasonable assumptions we can conclude that we have a global, qualitative understanding of this subspace. The first assumption is that Equation 2 provides a reasonable qualitative understanding of the model. If there are no bifurcations, the analysis presented here will identify the most influential parameters. When only two parameter values are tested it is not possible to pick nonlinearities in the initial experiment but subsequent experiments can be designed to pursue potentially interesting nonlinearities if a more quantitative understanding is required. If the model bifurcates this will be apparent in the output, for example as bimodal histograms or global deviations from normality in the quantile plot. By plotting histograms while holding individual parameters constant, it will be possible to identify the bifurcation parameter. It is worth noting that determining ranges of parameters, both for formal and *ad hoc* methods, must still be guided by physiological considerations and preliminary model exploration. Bifurcations are of immense interest as they indicate a switch from one behavior to another and the suspected bifurcations can be explored with further model runs. The second assumption is that high order interactions (combinations of 3 or more parameters) are not significant. The number of values estimated is equal to the number of runs less one. Most of these are estimates of higher order effects, yet we found that only first order and some second order effects were significant. This supports the assumption that higher effects do not contribute qualitatively to model behavior. We believe this and similar methods for gaining a global understanding of large parameter spaces will play an important role in brain modeling.

### Conflict of interest statement

The authors declare that the research was conducted in the absence of any commercial or financial relationships that could be constructed as a potential conflict of interest.
